# Web-based Guidance for Assisted Reproductive Technology With an Online App (myFertiCare): Quantitative Evaluation With the HOT-fit Framework

**DOI:** 10.2196/38535

**Published:** 2023-01-24

**Authors:** Ellen Marie Sparidaens, Jade G M Logger, Willianne L D M Nelen, Didi D M Braat, Kathrin Fleischer, Rosella P M Hermens

**Affiliations:** 1 Department of Obstetrics and Gynaecology Radboud University Nijmegen Medical Centre Nijmegen Netherlands; 2 Department of Dermatology Maastricht University Medical Center Maastricht Netherlands; 3 Scientific Institute for Quality of Healthcare Radboud University Medical Center Nijmegen Netherlands

**Keywords:** eHealth, personalized, interactive, evaluation, HOT-fit framework, assisted reproductive technologies, reproductive, technology, online, app, application, tool, internet, usability, infertility, variables, treatment, women, care, stress

## Abstract

**Background:**

Assisted reproductive technologies (ARTs) are considered to be physically and mentally stressful. During their treatment trajectory, couples express high information and communication needs. They appreciate using the internet to obtain fertility-related information. In a previous study, we developed myFertiCare, an eHealth tool providing personalized information and interactive functionalities for infertile couples in order to improve patient-centered care. The app has already been successful in qualitative evaluations of usability.

**Objective:**

The aim of the current study is to quantitatively evaluate the implementation of myFertiCare by using the human, organizational, and technology–fit (HOT-fit) framework and to study the effects of using myFertiCare on couples’ knowledge about infertility, their experience of the burden of infertility, and their experience of patient-centered care. With these results, implementation can be further improved, and patient-centered care can be enhanced.

**Methods:**

A quantitative study was performed based on the HOT-fit framework using validated questionnaires focusing on the *human, organizational*, and *technology* domains. Questions were added on the effect of using myFertiCare on couples’ knowledge about infertility and treatment. Questions regarding the burden of infertility, the burden of infertility treatment, and the experience of patient-centeredness were based on the main items of the validated fertility quality of life (FertiQoL) and Patient-Centredness Questionnaire–Infertility questionnaires, respectively. Also, nonusers of the app were included to explore motivations for not using the app and identify opportunities for improvement. Finally, user data were analyzed to provide insight into multiple variables concerning app use.

**Results:**

In the human and technology domains, myFertiCare showed good system usability, high user satisfaction, and high information and interface quality. In the organizational domain, implementation was considered to be sufficient by both patients and staff. Use of the app increased knowledge about the treatment, improved coping with the treatment, and enhanced the experience of patient-centeredness. User data showed that women were the main app users and that use of the app gradually declined during the treatment trajectory.

**Conclusions:**

A multi-faceted online app, myFertiCare, has been successfully evaluated quantitatively for implementation by using the HOT-fit framework. Use of the app increased knowledge about the treatment, improved coping with the treatment, and enhanced the experience of patient-centeredness. App use could be improved by creating more publicity. By providing myFertiCare, professionals in fertility care are supported in guiding patients through their treatment trajectory and in delivering patient-centered care.

## Introduction

Subfertility is defined as the failure to obtain a pregnancy after more than 12 months of unprotected intercourse [1]. It occurs in 1 of every 6 or 7 couples [2]. Depending on the results of diagnostic tests, assisted reproductive technologies (ARTs), such as in vitro fertilization (IVF) or intracytoplasmic sperm injection (ICSI), are available for these couples. ARTs are considered mentally and physically stressful [3]. Couples using ARTs are often young and well educated and want to be actively involved during their treatment [4]. As a result, these couples express high information and communication needs [4].

Subfertile couples appreciate using the internet to obtain fertility-related information [5]. Usually, the female partner is the main internet user. Most patients state that the internet improves their knowledge about infertility [5]. In practice, at least half of all subfertile couples use the internet for fertility-related purposes [5-8]. An online survey even characterized the internet as the most heavily relied upon source of information about infertility [9]. However, the quality of information provided on the internet is variable; it is often incomplete, misleading, or inaccurate [10,11]. Besides informational needs, other reasons for subfertile couples to use the internet are availability of emotional and social support and help with decision-making [11,12]. Patients prefer personal medical information online, such as access to their medical records, and interactive functionalities, such as the possibility to communicate with doctors and fellow patients [5]. They feel that online support from peers is helpful in dealing with emotional stress and social isolation [5,13].

As described in our previous study, we were the first to design and develop an online app (myFertiCare) for infertile patients that provides personalized information and interactive functionalities regarding their fertility treatment in order to improve the patient-centeredness of care [14]. We established the need for such an online app specifically among couples using ICSI with surgically retrieved sperm because of the psychological and physical burden of the multidisciplinary treatment. MyFertiCare provides personalized information and interactive functionalities as options for communication with doctors and fellow patients. The app has been successfully evaluated qualitatively for usability [14]. The aim of the current study is to quantitatively evaluate the implementation of the app by using the human, organizational, and technology–fit (HOT-fit) framework and to study the effects of using myFertiCare on couples’ knowledge about fertility treatment, their experience of treatment burden, and their experience of patient-centered care.

## Methods

### Study Design

We used a quantitative study design to evaluate the implementation of myFertiCare according to the HOT-fit framework. This framework states that a fit between human, organization, and technology factors is needed to successfully implement an eHealth intervention [15]. The HOT-fit framework was applied by using validated questionnaires focusing on these 3 domains. Furthermore, we studied the effect of using myFertiCare on (1) couples’ knowledge about fertility treatment, (2) couples’ experiences of treatment burden, and (3) couples’ experiences of patient-centered care. We also included nonusers of myFertiCare to explore motivations for not using the app and to identify opportunities for improvement. Finally, we analyzed user data to obtain insight into various app-related variables, such as the number of users, visits, and page views and the frequency and duration of use.

### Ethical Considerations

Ethical approval was proposed but was not required according to the local research ethics committee (Commissie Mensgebonden Onderzoek Arnhem Nijmegen).

### Setting

The study was established at a Dutch university medical center specializing in ART and surgical sperm retrieval for men with azoospermia. ICSI with surgical sperm retrieval is a multidisciplinary treatment involving a urologist, who is responsible for the andrological evaluation and surgical sperm retrieval; a gynecologist, who is responsible for the subsequent ICSI procedure; a psychologist, who is present for easily accessible mental support; and, if necessary, a clinical geneticist. In January 2016, the online app myFertiCare was launched and made available via the clinic’s website, the Apple App Store, and the Google Play Store.

### Participants

All couples visiting the outpatient clinic for possible ICSI with surgical sperm retrieval between January 1, 2016, and July 1, 2017, were invited to use myFertiCare and to participate in the questionnaire study. Men undergoing surgical sperm retrieval for fertility preservation purposes and couples in which neither partner understood the Dutch language were excluded. All participating couples signed an informed consent form.

### Data Collection

The study comprised 2 separate questionnaires: one was targeted at users of myFertiCare and one was targeted at nonusers. The questionnaires were available both on paper and digitally (using Castor electronic data capture). They were sent out in June 2017. Both questionnaires contained questions on the demographic and medical characteristics of the participants.

The user questionnaire was based on the principles of the HOT-fit framework [15]. The *human* domain consisted of system use and user satisfaction. Both were evaluated through validated questionnaires: the System Usability Scale (SUS) [16] and the End-User Computing Satisfaction (EUCS) instrument [17], respectively. The *organization* domain consisted of the structural and environmental context of fertility care and was evaluated through self-developed questions in the user questionnaire (a validated questionnaire was not used because the questions were unique to the context of the organization) and through short, face-to-face structured interviews with staff members of the department of reproductive medicine. The interview questions focused on the staff’s experiences with the organization and implementation of myFertiCare. These interviews were performed face-to-face because this revealed more subtle motivations. Relevant items regarding the implementation of myFertiCare were identified and reported as results. The *technological* domain included the quality of the system, information, and service (ie, the interface). This was studied through the *information* and *interface quality* domains of the validated Computer System Usability Questionnaire (CSUQ) [18]. All validated questionnaires were translated into Dutch by a member of the research team. The translations were checked by having another member translate them back into English. Both researchers were proficient in English and Dutch. Discrepancies were discussed until consensus was reached. To supplement the HOT-fit framework, questions were added concerning knowledge about infertility, the burden of infertility and fertility treatment, and the extent to which couples experienced patient-centered care. Couples were asked if using myFertiCare increased their knowledge about the causes of and treatments for infertility. Questions concerning the burden of infertility and infertility treatment were based on the main items described in the validated fertility quality of life (FertiQoL) questionnaire [19]. Questions about patient-centeredness were based on the 8 subheadings of the validated Patient-Centredness Questionnaire–Infertility [20]. The complete user questionnaire consisted of 72 questions with answer options being open, on a 5- or 7-point Likert scale, or on a unipolar verbal scale.

The questionnaire for nonusers of myFertiCare consisted of 4 questions regarding familiarity with the availability of myFertiCare, motivations for not using the app, and suggestions to increase its use and the use of other online sources of information. The questions were self-developed by the research team to match the specific context of the organization.

Both questionnaires were pilot-tested with 3 couples attending the outpatient clinic. The couples considered the questions clear and understandable, so no major changes were necessary. The questionnaires were sent out via postal mail by a member of the treatment team. One questionnaire was sent per couple, addressed to the couple, because a close connection and interaction between partners during the treatment was assumed. Each couple received both the user and nonuser questionnaires and had to decide which of the two was applicable. Use was defined as the minimum of one log-in to the app; nonuse was defined as never having logged in. Patients had to write down who completed the questionnaire: the male partner, the female partner, or both. Couples could return the questionnaire via postal mail or email. Nonresponders were sent a reminder 2 weeks after the initial invitation. Questionnaires were collected from July 1, 2017, until August 18, 2017, and assigned a code that was only available to the researchers. All data were analyzed confidentially.

User data were automatically transferred to an anonymized Microsoft Excel file. In this way, all app visits and page views could be logged by date and time and analyzed later. All patients were included who used myFertiCare between January 1, 2016, and July 1, 2017, whether they participated in the questionnaire study or not.

### Data Analysis

Statistical analysis was performed using Microsoft Excel and SPSS (version 22). Baseline characteristics of the study population, user data, and the results of the questionnaires were analyzed using descriptive statistics: median (range), mean (SD), or frequency. We also focused on finding possible differences in user data between men and women. *P* values were calculated using independent-sample 2-tailed *t* tests, Mann-Whitney *U* tests, and chi-square tests to find possible significant differences (*P*<.05) between users and nonusers and between men and women.

## Results

### Study Population

**Figure 1:**
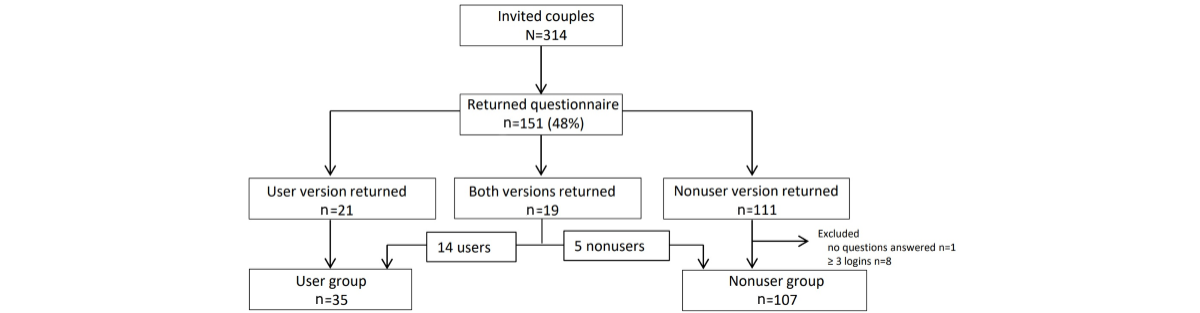
Overview of the participating couples.

In total, 314 ICSI couples were invited to participate (Figure 1); 151 couples (48%) completed and returned a questionnaire, including 21 user questionnaires and 111 nonuser questionnaires. Additionally, 19 couples returned both versions of the questionnaire. If both versions were returned, the user data were consulted to classify these couples as users or nonusers and the appropriate questionnaire was included in the analysis. Nine nonuser questionnaires were excluded because no questions were filled in or because the couples actually were users, which was defined as 3 or more log-ins. Finally, the user group consisted of 35 couples and the nonuser group of 107 couples (Figure 1). Demographic and medical characteristics of the participants (users and nonusers) are provided in Tables 1 and 2.

**Table 1:** Demographic and clinical characteristics of men and women in the study, both users and nonusers.

Characteristics					Users (n=35 couples)		Nonusers (n=107 couples)		*P* value
**Men**									
	Age (years), mean (SD)				37 (7.7)		37 (7.6)		.80^a^
	Dutch background, n (%)				35 (100)		103 (96)		.57^b^
	**Educational status^c^, n (%)**								.44^d^
		Low		6 (18)		10 (10)			
		Medium		12 (36)		40 (40)			
		High		15 (46)		51 (50)			
	**Parental status, n (%)**								.47^e^
			Children	12 (34)		44 (41)			
			No children	23 (66)		63 (59)			
**Women**									
	Age (years), mean (SD)				32 (4.3)		32 (4.0)		.51^a^
	Dutch background, n (%)				32 (91)		103 (96)		.36^b^
	**Educational status^c^, n (%)**								.60^d^
		Low		2 (6)		5 (5)			
		Medium		13 (37)		48 (47)			
		High		20 (57)		50 (48)			
	**Parental status, n (%)**								.86^e^
			Children	12 (34)		35 (33)			
			No children	23 (66)		72 (67)			
	Currently pregnant				7 (20)		31 (29)		.48^e^

^a^Obtained with an independent-sample *t* test.

^b^Obtained with the Fisher exact test.

^c^Educational status: Low educational status included no education and lower general secondary education; medium educational status included higher general secondary education and intermediate vocational education; high educational status included higher vocational education and a university degree.

^d^Obtained with the Fisher-Freeman-Halton test.

^e^Obtained with the chi-square test.

**Table 2:** Demographic and clinical characteristics of couples, both users and nonusers.

Characteristics		Users (n=35 couples)	Nonusers (n=107 couples)	*P* value	
**Socioeconomic status^a^, n (%)**					.24^b^
	Low	11 (31)	19 (18)		
	Medium	21 (60)	78 (73)		
	High	3 (9)	10 (9)		
Duration of infertility (months), median (range)		24 (8-120)	30 (2-120)	.05^c^	
**Current stage of infertility treatment, n (%)**					N/A^d^
	Out of treatment^e^	17 (49)	80 (75)		
	Before surgical sperm retrieval^f^	1 (3)	3 (3)		
	After surgical sperm retrieval, before ICSI^g,h^	2 (6)	3 (3)		
	During first ICSI cycle^i^	6 (17)	7 (7)		
	During a following ICSI cycle or cryo cycle	9 (26)	14 (13)		

^a^Classified according to the Dutch Social and Cultural Planning Office definitions: low socioeconomic status was a status score of ≤–1; medium socioeconomic status was a status score between –1 and 1; high socioeconomic status was a status score of >1.

^b^Obtained with the chi-square test.

^c^Obtained with the Mann-Whitney test.

^d^N/A: not applicable; not calculated because of small subgroups.

^e^These couples were either pregnant or had exhausted all treatment options.

^f^These couples were undergoing the diagnostic process before surgical sperm retrieval.

^g^ICSI: intracytoplasmic sperm injection.

^h^The results of surgical sperm retrieval were being evaluated in preparation for ICSI.

^i^These couples were starting the first ICSI cycle; no pregnancy test had yet been adminstered.

### User of myFertiCare

We analyzed questionnaires from 35 couples who were myFertiCare users according to the HOT-fit framework.

#### Human and Technology Domains

In 42% (13/31) of the couples, the female partner was the only user of myFertiCare. In 19% (6/31), only the male partner used the app. In 39% (12/31) of the couples, both partners used the app, all of whom had at some point used the app together; in 92% (11/12) of these couples, the female partner was the main user.

The results of the 3 validated questionnaires for the *human* and *technology* domains (the SUS and EUCS, both of which examined the *human* domain, and the CSUQ, which examined the *technology* domain) are shown in Table 3. The mean SUS score was 73, which implies good system usability (the scale ranges from 0 to 100, with 100 being the best possible score) [21]. All subitems of the EUCS showed high user satisfaction (the total score was 47, on a scale from 12 to 60, with 60 being the best possible score) [17]. The CSUQ showed high information quality (the score was 18 on a scale from 7 to 49, with 7 being the best possible score) and interface quality (the score was 9 on a scale from 3 to 21, with 3 being the best possible score) [18].

Users stated that they would recommend myFertiCare to a friend (25/26, 96%) because they considered the app to be informational and easy to handle. They stated they would use similar apps if they were available when visiting other medical departments (25/26, 96%). The most appreciated functionality was the visualized treatment trajectory, which showed the couple’s scheduled and as-yet unscheduled appointments on a visual timeline. Some couples gave suggestions for future app functionalities, such as a medication schedule and a mood tracker. The couples were confident that the app safeguarded their personal information (25/26, 96%). Suggestions to achieve more frequent app use included increasing publicity for the app, increasing activity on the forum, and using the app during outpatient appointments.

**Table 3:** Scores for the System Usability Scale, End-User Computing Satisfaction, and Computer System Usability Questionnaire scales of users of myFertiCare.

Questionnaire			Response rate^a^, n		Median score (range)		Possible range of scores
System Usability Scale (higher scores are better)			25		73 (43-93)		0-100
**End-User Computing Satisfaction (higher scores are better)**							
	Content	24		15 (4-19)		4-20	
	Accuracy	23		8 (2-10)		2-10	
	Format	23		8 (2-10)		2-10	
	Ease of use	24		8 (2-10)		2-10	
	Timelines	22		8 (2-10)		2-10	
	Total	21		47 (12-59)		12-60	
**Computer System Usability Questionnaire (lower scores are better)**							
	Information quality	22		18 (6-36)		7-49	
	Interface quality	22		9 (3-20)		3-21	

^a^The response rate was defined because not every couple (n=35) answered every question.

#### Organization Domain

The couples were asked how they found out about the availability of myFertiCare. Most of them remembered being informed verbally or in writing by a member of the treatment team (25/32, 78%). A minority did not remember being informed, but said they found the app on the hospital website (3/32, 9%). Information about the app was considered complete and well timed. Couples who could not remember being informed about the availability of myFertiCare stated they would have appreciated this. The majority of couples felt that myFertiCare was well used in fertility care (23/28, 82%) and that the treatment team was sufficiently familiar with the app (19/25, 76%). One third of couples were not aware of whom to ask questions to about the app (10/28, 36%). All couples that did ask a question about the app to a member of the treatment team stated they received a satisfying answer.

The *organization* domain was also studied through interviews with members of the treatment team (n=17). We interviewed 3 fertility doctors, 3 nurses, 3 medical assistants, 3 secretaries, 2 gynecologists, 1 laboratory employee, 1 embryologist, and 1 operational manager. All team members were aware of the availability of myFertiCare and had been informed about myFertiCare by a presentation from the researchers or via email. They described the app with the following terms: “digital support,” “information about treatment trajectory and appointments,” “timeline,” “finding advice and test results,” “chat function with peers,” and “asking questions to the treatment team.” Ten of the staff did not know if patients were sufficiently aware of the functionalities of myFertiCare and 5 of them wanted to know more about the functionalities themselves. All team members were aware of whom to contact in case they had questions about or problems with myFertiCare. Furthermore, all team members recommended using myFertiCare to patients. Use of the app could be improved by creating more awareness, expanding its use to other ART treatments, maintaining up-to-date information, and evaluating its use during outpatient appointments.

#### Effects of App Use

To supplement the HOT-fit evaluation, we studied the effect of using myFertiCare on (1) knowledge about infertility and treatment, (2) the burden of infertility and treatment, and (3) the patient-centeredness of care.

##### Knowledge About Infertility and Treatment

Half the couples reported that using myFertiCare did not increase at all or only slightly increased knowledge about the causes of infertility. However, 79% did feel that myFertiCare increased knowledge about fertility treatment (Table 4).

**Table 4:** Effect of myFertiCare on knowledge.

Respondents, n	myFertiCare has increased my knowledge about the causes of infertility	myFertiCare has increased my knowledge about the treatment of reduced fertility
Not at all	11	5
Slightly	3	1
Moderately	8	5
Strongly	6	11
Very strongly	2	7

##### Burden of Infertility and Treatment

**Figure 2:**
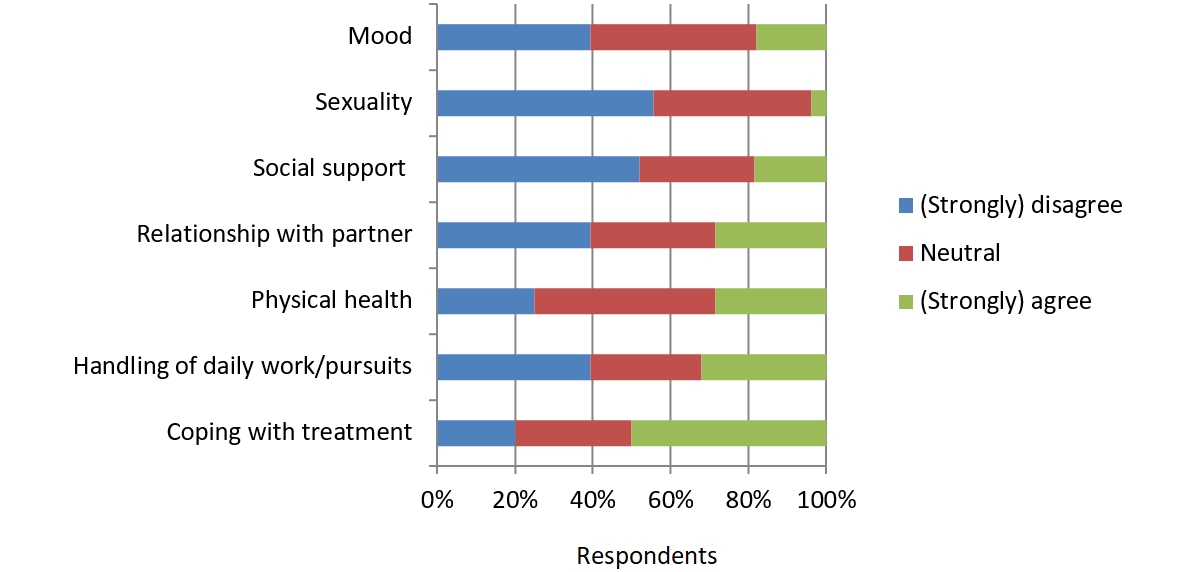
Effect of myFertiCare on the burden of infertility and treatment. The respondents were asked to complete the following sentence: “myFertiCare positively contributed to my...”.

Half the couples indicated that myFertiCare contributed positively to coping with treatment (Figure 2). We observed a tendency for respondents to be neutral about or deny the possibility that myFertiCare had an effect on their handling of daily activities, physical health, relationship with their partner, social support, sexuality, and mood (Figure 2). The general opinion was that myFertiCare was mainly a source of information and not a tool that significantly influenced the burden of infertility and treatment.

##### Patient-Centeredness of Care

**Figure 3:**
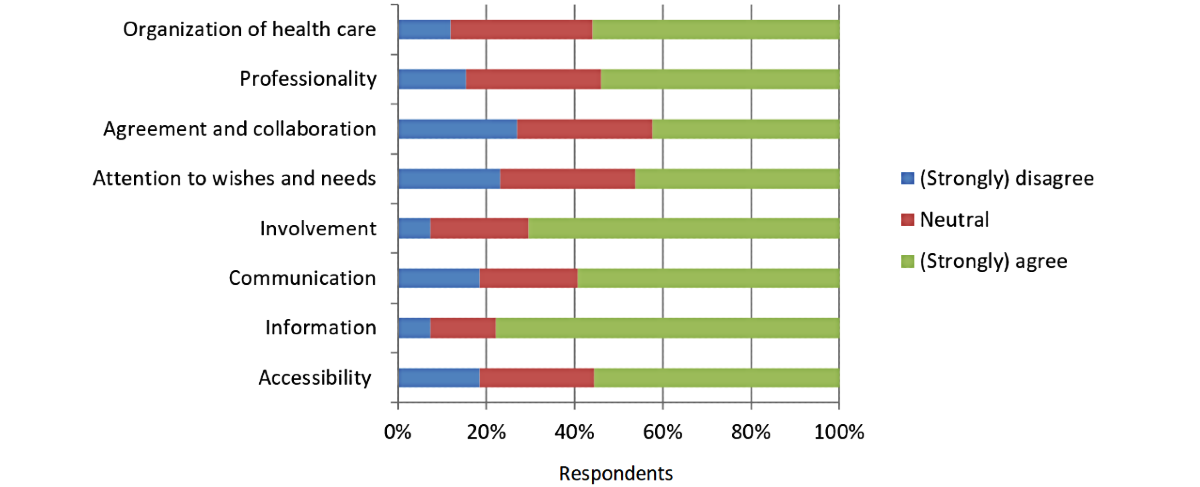
Effect of myFertiCare on the experience of patient-centeredness of care. The respondents were asked to complete the following sentence: “myFertiCare increased...”.

The couples were positive about the effect of myFertiCare on their experience of patient-centered fertility care. All 8 surveyed items (ie, accessibility, information, communication, involvement, attention to wishes and needs, agreement and collaboration, professionality, and organization of health care) were improved by the use of myFertiCare (Figure 3).

### Nonusers of myFertiCare

Questionnaires from 107 couples who were nonusers of myFertiCare were analyzed. Although providing information about myFertiCare was part of routine care, only 25% (27/107) of nonusers remembered being informed about the availability of myFertiCare. The main reason for nonuse was a lack of need or interest. Of the couples that did not remember being informed about myFertiCare, half (54/107) said that they would have liked to use the app. The other half felt that they did not need the app because they obtained sufficient information via other sources. The majority of nonusers of myFertiCare used other sources of online information about infertility or treatment (71/107, 66%).

### User Data

We analyzed user data as a supplement to the questionnaire study. In total, 163 individual patients used myFertiCare, who were included in 139 couples. In 21% (29/139) of couples, only the male partner used the app, while in 47% (66/139) of couples, only the female partner used the app, and in 32% (44/139) of couples, both partners used the app. When both partners were users, the female partners were the more frequent users, with a median of 9 visits, compared to a median of 3 visits for the male partners. All user data are shown in Table 5. Finally, it appeared that use of myFertiCare gradually declined during the treatment trajectory, as illustrated in Table 6.

**Table 5:** User data for myFertiCare from December 8, 2015, until August 4, 2017.

User data	Total (n=163)	Men (n=61)	Women (n=102)	*P* value
Visits, n	951	192	759	N/A^a^
Page views, n	17,097	3734	13,363	N/A
One-visit-users, n (%)	51 (31)	25 (41)	26 (26)	.04^b^
Visits per user, median (range)	3 (1-85)	2 (1-23)	3 (1-85)	.001^c^
Frequency of visits (interval between visits in days), mean	5.4	12	3.6	.006^c^
Duration per visit^d^ (minutes), median (range)	1.03 (0-107)	1.55 (0-107)	0.93 (0-51)	.12^c^
Time between first and last visit (days), median (range)	30 (1-499)	63 (1-499)	20 (1-347)	.01^c^
Total duration of use (minutes), median (range)	9.6 (0-268)	5.9 (0-119)	13 (0-268)	.01^c^
Page views per user^e^, median (range)	59 (0-1254)	47 (0-375)	76 (1-1254)	.002^c^
Page views per visit^e^, median (range)	11 (0-139)	10 (0-88)	11 (0-139)	0.96^c^

^a^N/A: not applicable.

^b^Obtained with chi-square test.

^c^Obtained with Mann-Whitney test.

^d^Users were logged out automatically after 20 minutes of inactivity.

^e^Excluding view of navigation pages (ie, log-in or log-out pages).

**Table 6:** Use of myFertiCare per treatment phase. The phases included (1) before surgical sperm retrieval; (2) after surgical sperm retrieval, during preparation for ICSI^a^; (3) during ICSI treatment, but before visits to the outpatient clinic; (4) during ICSI treatment, between the first and last visits to the outpatient clinic; (5) during ICSI treatment, between the embryo transfer and the pregnancy test; and (6) after ICSI. Phase 6 included (6A) the period between a negative pregnancy test or cancellation of treatment until the start of a new treatment; (6B) the period between a positive pregnancy test and the first ultrasound; (6C) pregnancy, during the period after the first ultrasound; and (6D) exhaustion of all treatment options (without pregnancy).

	Treatment phase								
	1	2	3	4	5	6A	6B	6C	6D
App users, n	90	70	47	39	26	26	14	11	12
Page views, n	7260	3345	1791	1856	789	828	459	237	253

^a^ICSI: intracytoplasmic sperm injection.

## Discussion

### Principal Results

In this quantitative study, the implementation of myFertiCare was evaluated with the HOT-fit framework. In the *human* and *technology* domains, myFertiCare showed good system usability, high user satisfaction, and high information and interface quality. In the *organizational* domain, based on high scores in the user questionnaire and positive feedback from the staff interviews, implementation was considered to be sufficient. App use could have been improved by creating more awareness among patients and staff. Use of the app increased knowledge about the treatment, improved coping with the treatment, and enhanced the experience of patient-centeredness of care.

The current study shows that women were the main app users. Either the female partner was the only user or was the main user when both partners used the app. This observation is in agreement with previous research [5,7,8,22]. It is attributable to sex differences in health-related internet use, the experience of infertility and fertility treatment, and strategies to cope with fertility-related issues [23]. However, we found that although men were less frequent myFertiCare users, the duration and number of page views per visit was equal for both sexes.

User data showed that use of the app was highest before surgical sperm retrieval and gradually declined thereafter. The observed gradual decline in app use contradicts previous research, which found that the highest user activity occurred in later treatment phases, namely between oocyte retrieval, embryo transfer, and the pregnancy test [24], which was attributed to high stress levels during this particular treatment phase [3,25]. This could be explained by the law of attrition, which is a phenomenon whereby participants discontinue use of eHealth interventions that are neither mandatory nor critical to their direct well-being [26]. However, a part of this decline was also caused by the expected treatment dropout that occurs with a negative result from the surgical sperm retrieval. It is known that in half of men with nonobstructive azoospermia, no sperm cells can be retrieved [27]. Therefore, these couples were not able to continue their fertility treatment with an ICSI procedure. Their treatment stopped, as did their use of myFertiCare. Furthermore, as a result of a relatively high rate of fertilization failure in our study related to the use of surgically retrieved sperm instead of the use of ejaculated sperm [28], a proportion of our participants probably did not proceed to embryo transfer and therefore did not use the app at this treatment stage. On the other hand, the couples that continued with ICSI treatment were possibly satisfied with the information they received, and the need for app use declined.

It is remarkable that the majority of nonusers of the app did not remember being informed about the availability of myFertiCare. All couples received this information as part of standard care during an informative group consultation conducted by a specialized nurse at the beginning of the treatment trajectory. It is known that patients’ memory for medical information is often poor and inaccurate, especially when the patient is anxious [29]. This underlines the necessity of repeating important information on several occasions and providing information in written form.

By studying the effects of app use, we found that users considered myFertiCare to be mainly a source of information, rather than a tool able to significantly decrease the burdens of infertility and treatment. However, the couples were outspoken that myFertiCare improved their experience of patient-centeredness of care, meeting the goal we set at the start of the study. Therefore, myFertiCare constitutes an innovative tool to help professionals provide patient-centered care. We hypothesize that myFertiCare could score higher for influence on the burdens of infertility and treatment if it were supplemented with functionalities targeting this effect. In the current study, we developed an online app for a pilot population and were not equipped with resources to add extra functionalities. Another possible way of improving app use would be to develop an app that also provides a benefit for the treatment team, such as by making it easier or more efficient to provide care to patients or by making that care better. Therefore, we call for other medical professionals to continue developing online interventions in collaboration with their patients and staff, so that patient-centered care can be improved from the perspective of the patient and the professional.

Numerous eHealth interventions with different functionalities targeted at a variety of patient categories have been reported. It is remarkable that most evaluations of these interventions are only performed at the end of the intervention, although the importance of conducting evaluations throughout an intervention is regularly discussed [30]. A study of eHealth evaluations made the striking finding that only 64% of studies evaluated clinical aspects, 48% evaluated human and social aspects, 20% evaluated technological aspects, and 16% evaluated organizational aspects [30]. A recent example of an eHealth intervention that did include human, organization, and technology factors in the evaluation used a qualitative study design with semistructured interviews to explore patients’ experiences and described these experiences on the basis of the 3 domains [31]. Therefore, we feel that the major strength of the current study is the study design. By using validated questionnaires on the human, organizational, and technological domains, we quantitatively studied implementation of the app. Furthermore, we studied the effects of app use and analyzed user data. Finally, we also included nonusers of the app in the study, to explore motivations for not using the app and identify opportunities for improvement. This way, we provided a complete framework for app design, development, implementation, evaluation, and improvement. We call for better evaluation of eHealth interventions to facilitate successful long-term implementation.

Our study also has limitations. There was a relatively low response rate of 25% (35 of 139 couples) to the user questionnaires. For the nonuser questionnaires, the response rate was 61% (107 of 175 couples). A possible explanation could be that we sent the questionnaires in June 2017 to all couples who were treated between January 2016 and July 2017. It could be that a significant proportion of these couples had already dropped out or finished treatment. Another hypothesis is that the response rate could have been affected by the length of the questionnaires. Because we used multiple validated questionnaires on different domains, the user questionnaire was quite extensive, whereas the nonuser questionnaire contained only 4 questions. However, there was good consistency in the data from the user questionnaires, which supports the reliability of the study data. Another limitation is that we studied the effects of app use (ie, knowledge about infertility and treatment, the burdens of infertility and treatment, and patient-centeredness of care) based on self-reported differences before and after app use, rather than measurements before and after app use. We chose this approach because we did not want patients to feel that they had to perform an exam instead of a questionnaire, and we did not want to make the questionnaire more extensive to study patients’ knowledge.

### Conclusion

A multi-faceted online app, myFertiCare, has been successfully evaluated quantitatively for implementation with the HOT-fit framework. Use of the app increased knowledge about the treatment, improved coping with the treatment, and enhanced the experience of patient-centeredness of care.

### Practice Implications

In our previous study, we successfully designed, developed, and qualitatively evaluated myFertiCare for usability. In the current study, implementation of the app was positively and quantitatively evaluated based on the HOT-fit framework, and the effects of app use were studied. Through these consecutive studies, a framework has become available that can be used throughout the complete trajectory of app development, implementation, evaluation, and improvement, and which involves both patients and professionals in every study phase.

Providing myFertiCare encourages professionals in fertility care to guide patients through their treatment trajectory and to deliver patient-centered care. Furthermore, myFertiCare offers an opportunity to empower patients and help them manage their own treatment trajectories. It would be valuable for future research to improve the app based on the couples’ and professionals’ suggestions, so that more support is perceived and app use can be expanded to other patient categories and medical departments. We appeal to professionals in both fertility care and other medical departments to provide eHealth initiatives to their patients in which both patients and professionals are involved in every phase of design, development, implementation, and evaluation.

## Abbreviations

ART: assisted reproductive technology
CSUQ: Computer System Usability Questionnaire
EUCS: End-User Computing Satisfaction
HOT-fit: human, organization, and technology–fit
ICSI: intracytoplasmic sperm injection
IVF: in vitro fertilization
SUS: System Usability Scale
